# Solving Set Cover with Pairs Problem using Quantum Annealing

**DOI:** 10.1038/srep33957

**Published:** 2016-09-27

**Authors:** Yudong Cao, Shuxian Jiang, Debbie Perouli, Sabre Kais

**Affiliations:** 1 Department of Computer Science, Purdue University, West Lafayette, IN 47906, USA; 2 Department of Mathematics, Statistics and Computer Science, Marquette University, Milwaukee, WI 53233, USA; 3 Department of Chemistry, Physics and Birck Nanotechnology Center, Purdue University, West Lafayette, IN 47906, USA; 4 Qatar Energy and Environment Research Institute (QEERI), HBKU, Doha, Qatar

## Abstract

Here we consider using quantum annealing to solve Set Cover with Pairs (SCP), an NP-hard combinatorial optimization problem that plays an important role in networking, computational biology, and biochemistry. We show an explicit construction of Ising Hamiltonians whose ground states encode the solution of SCP instances. We numerically simulate the time-dependent Schrödinger equation in order to test the performance of quantum annealing for random instances and compare with that of simulated annealing. We also discuss explicit embedding strategies for realizing our Hamiltonian construction on the D-wave type restricted Ising Hamiltonian based on Chimera graphs. Our embedding on the Chimera graph preserves the structure of the original SCP instance and in particular, the embedding for general complete bipartite graphs and logical disjunctions may be of broader use than that the specific problem we deal with.

Quantum annealing (QA) uses the principles of quantum mechanics for solving unconstrained optimization problems^1^,^2^,^3^,^4^. Since the initial proposal of QA, there has been much interest in the search for practical problems where it can be advantageous with respect to classical algorithms^4^,^5^,^6^,^7^,^8^,^9^,^10^,^11^,^12^,^13^,^14^,^15^,^16^,^17^,^18^,^19^,^20^,^21^,^22^,^23^,^24^,^25^,^26^,^27^,^28^,^29^,^30^,^31^,^32^,^33^, particularly simulated annealing (SA)^34^,^35^,^36^. Extensive theoretical, numerical and expeirmental efforts have been dedicated to studying the performance of quantum annealing on problems such as satisfiability^37^,^38^,^39^, exact cover^3^,^39^, max independent set^39^, max clique^40^, integer factorization^41^, graph isomorphism^42^,^43^, ramsey number^44^, binary classification^45^,^46^, unstructured search^47^ and search engine ranking^48^. Many of these approaches^3^,^37^,^38^,^40^,^41^,^42^,^43^,^44^,^45^,^46^ recast the computational problem at hand into a problem of finding the ground state of a quantum Ising spin glass model, which is NP-complete to solve in the worst case^49^,^50^.

The computational difficulty of Ising spin glass has not only given the quantum Ising Hamiltonians the versatility for efficiently encoding many problems in NP^50^, but also motivated physical realization of QA using systems described by the quantum Ising model^6^,^7^,^9^. The notion of adiabatic quantum computing (AQC)^3^,^37^,^51^, which can be regarded as a particular class of QA, has further established QA in the context of quantum computation (In this work we will use the terms quantum annealing and adiabatic quantum computing synonymously). Although it is believed that even universal quantum computers cannot solve NP-hard problems efficiently in general^52^, there has been evidence in experimental quantum Ising systems that suggests quantum speedup over classical computation due to quantum tunneling^53^,^54^. It is then of great interest to explore more regimes where quantum annealing could offer a speedup compared with simulated annealing.

Here we consider a variant of Set Cover (SC) called Set Cover with Pairs (SCP). SC is one of Karp’s 21 NP-complete problems^55^ and SCP was first introduced^56^ as a generalization of SC. Instead of requiring each element to be covered by a single object as in SC, the SCP problem is to find a minimum subset of objects so that each element is covered by *at least one pair* of objects. We will present its formal definition in the Preliminaries section. SCP and its variants arise in a wide variety of contexts including Internet traffic monitoring and content distribution^57^, computational biology^58^,^59^, and biochemistry^60^. On classical computers, the SCP problem is at least as hard to approximate as SC. Specifically, its difficulty on classical computers can be manifested in the results by Breslau *et al*.^57^, which showed that no polynomial time algorithm can approximately solve Disjoint-Path Facility Location, a special case of SCP, on *n* objects to within a factor that is 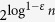 for any *ε* > 0. Due to its complexity, various heuristics^56^ and local search algorithms^60^ have been proposed.

In this paper we explore using quantum annealing based on Ising spin glass to solve SCP. We start by reducing SCP to finding the ground state of Ising spin glass, via integer linear programming (Theorem 1). We then simulate the adiabatic evolution of the time dependent transverse Ising Hamiltonian 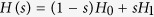 which interpolates linearly between an initial Hamiltonian *H*_0_ of independent spins in uniform transverse field and a final Hamiltonian *H*_1_ that encodes an SCP instance. For randomly generated SCP instances that lead to Ising Hamiltonian constructions of up to 19 spins, we explicitly simulate the time dependent Schrödinger equation. We compute the minimum evolution time that each instance needed to accomplish 25% success probability. For benchmark purpose we also use simulate annealing to solve the instances and compare its performance with that of adiabatic evolution. Results show that the median time for yielding 25% success probablity scales as *O*(2^0.33*M*^) for quantum annealing and *O*(2^0.21*M*^) for simulated annealing, observing no general quantum speedup. However, the performance of quantum annealing appears to have wider range of variance from instance to instance than simulated annealing, casting hope that perhaps certain subsets of the instance could yield a quantum advantage over the classical algorithms.

Aside from the theoretical and numerical studies, we also consider the potential implementation our Hamiltonian construction on the large-scale Ising spin systems manufactured by D-Wave Systems^6^,^7^,^9^,^14^. Benchmarking the efficiency of QA is currently of significant interest. An important issue that needs to be addressed in such benchmarks is that the physical implementation of the algorithm could be affected by instance-specific features. This is manifested in the embedding^61^,^62^ of the Ising Hamiltonian construction onto the specific topology of the hardware (the Chimera graph^21^,^61^,^63^). Here we present a general embedding of SCP instances onto a Chimera graph that preserves the original structure of the instances and requires less qubits than the usual approach by complete graph embedding. This allows for efficient physical implementations that are untainted by ad hoc constructions that are specific to individual instances.

## Preliminaries

### Set Cover with Pairs

Given a *ground set U* and a collection *S* of subsets of *U*, which we call the *cover set*. Each element in *S* has a non-negative weight, the Set Cover (SC) problem asks to find a minimum weight subset of *S* that covers all elements in *U*. Define *cover function* as 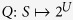 where 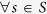, *Q*(*s*) is the set of all elements in *U* covered by *s*. Then SC can be formulated as finding a minimum weight 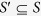 such that 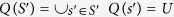. Set Cover with Pairs (SCP) can be considered as a generalization of SC in the sense that if we define the cover function such that 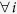, 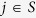, *i*≠*j*, *Q*(*i*, *j*) is the set of elements in *U* covered by the pair {*i*, *j*}, then SCP asks to find a minimum subset 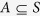 such that 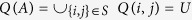. Here we restrict to cases where each element of *S* has unit weight.

A *graph G*(*V*, *E*) is a set of vertices *V* connected by a set of edges *E*. A *bipartite graph* is defined as a graph whose set of vertices *V* can be partitioned into two disjoint sets *V*_1_ and *V*_2_ such that no two vertices within the same set are adjacent. We formally define SCP as the following.

**Definition 1.** (*Set Cover with Pairs*) *Let U and S be disjoint sets of elements and* 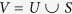. *Given a bipartite graph G*(*V*, *E*) *between U and S with E being the set of all edges*, *find a subset* 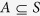 *such that*:

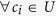
, 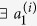, 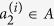 *such that* 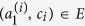 *and* 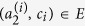. *In other words*, *c*_*i*_ *is covered by the pair* 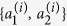.
2. *The size of the set*, |*A*|, *is minimized*.

*We use the notation* SCP(*G*, *U*, *S*) *to refer to a problem instance with* |*U*| = *n*, |*S*| = *m and the connectivity between U and S determined by G*.

### Quantum annealing, adiabatic quantum computing

In this paper we use QA as a heuristic method to solve the SCP problem. QA was proposed^2^ for solving optimization problems using quantum fluctuations, known as quantum tunneling, to escape local minima and discover the lowest energy state. Farhi *et al*.^3^ provide the framework for using Adiabatic Quantum Computation (AQC), which is closely related to QA, as a quantum paradigm to solve NP-hard optimization problems. The first step of the framework is to define a Hamiltonian *H*_*P*_ whose ground state corresponds to the solution of the combinatorial optimization problem. Then, we initialize a system in the ground state of some beginning Hamiltonian *H*_*B*_ that is easy to solve, and perform the adiabatic evolution 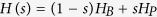. Here 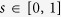 is a time parameter. In this paper we only consider time-dependent function *s*(*t*) = *t*/*T* for total evolution time *T*, but in general it could be any general functions that satisfy *s*(0) = 0 and *s*(*T*) = 1. The adiabatic evolution is governed by the Schrödinger equation


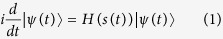


where |*ψ*(*t*)〉 is the state of the system at any time 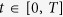. Let *π*_*i*_(*s*) be the *i*-th instantaneous eigenstate of *H*(*s*). In other words, let 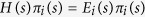 for any *s*. In particular, let |*π*_0_(*s*)〉 be the instantaneous ground state of *H*(*s*).

According to the adiabatic theorem^64^, for *s* varying sufficiently slow from 0 to 1, the state of the system |*ψ*(*t*)〉 will remain close to the true ground state |*π*_0_(*s*(*t*))〉. At the end of the evolution the system is roughly in the ground state of *H*_*P*_, which encodes the optimal solution to the problem. If the ground state of *H*_*P*_ is NP-complete to find (for instance consider the case for Ising spin glass^49^), then the adiabatic evolution *H*(*s*) could be used as a heuristic for solving the problem.

An important issue associated with AQC is that the adiabatic evolution needs to be slow enough to avoid exciting the system out of its ground state at any point. In order to estimate the scaling of the minimum runtime *T* needed for the adiabatic computation, criteria based on the minimum gap between the ground state and the first excited state of *H*(*s*) is often used. However, here we do not use the minimum gap as an intermediate for estimating the runtime scaling, but instead numerically integrate the time dependent Schrödinger equation (1).

### Quantum Ising model with transverse field

The Hamiltonian for an Ising spin glass on *N* spins can be written as


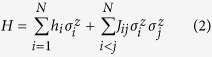


where 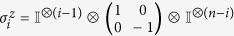 acts on the *i*-th spin with 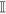 being a 2 × 2 identity matrix. *h*_*i*_, *J*_*ij*_ are coefficients. The Hamiltonian is diagonal in the basis 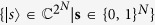 in the Hilbert space 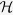. In particular *σ*^*z*^|0〉 = |0〉 and *σ*^*z*^|1〉 = −|1〉. We formally define the problem of finding the ground state of an *N*-qubit Ising Hamiltonian in the following.

**Definition 2.** (*Ising Hamiltonian*) *Given the Hamiltonian H in* equation (2), *find a quantum state* 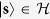, *where* 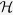 *is* 2^*N*^-*dimensional*, *such that the energy* 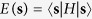 *is minimized*. *We use the notation* ISING (**h**, **J**) *to refer to the problem instance where* 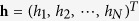 *and* 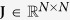 *is a matrix such that the ij*-*th and the ji*-*th elements are equal to J*_*ij*_/2. *The diagonal elements of* **J** *are 0*. *Hence* 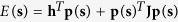 *where* 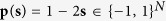.

In this paper, we construct Ising Hamiltonians whose ground state encodes the solution to an arbitrary instance of the SCP problem. The physical system used for quantum annealing that we assume is identical to that of D-Wave^6^,^7^,^9^,^14^, namely Ising spin glass with transverse field


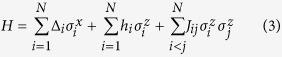


where 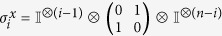 acts on the *i*-th spin. The beginning Hamiltonian *H*_*B*_ has its *h*_*i*_, *j*_*ij*_ = 0 for all *i*, *j* and the final Hamiltonian *H*_*P*_ has Δ_*i*_ = 0 for all *i* while *h*_*i*_ and *J*_*ij*_ depend on the problem instance at hand. We will elaborate on assigning *h*_*i*_ and *J*_*ij*_ coefficients in *H*_*P*_ in Theorem 1.

### Graph minor embedding

The interactions described by the transverse Ising Hamiltonian in equation (3) are not restricted by any constrains. However, in practice the topology of interactions is always constrained to the connectivity that the hardware permits. Therefore in order to physically implement an arbitrary transverse Ising Hamiltonian, one must address the problem of embedding the Hamiltonian into the logical fabric of the hardware^61^,^62^. For convenience we define the *interaction graph* of an Ising Hamiltonian *H* of the form in equation (2) as a graph *G*_*H*_(*V*_*H*_, *E*_*H*_) such that each spin *i* maps to a distinctive element *v*_*i*_ in *V*_*H*_ and there is an edge between *v*_*i*_ and *v*_*j*_ iff *J*_*ij*_≠0. This definition also applies to the transverse Ising system described in equation (3). We use the term *hardware graph* to refer to a graph whose vertices represent the qubits in the hardware and the edges describe the allowed set of couplings in the hardware.

In Section Set Cover with Pairs we defined bipartite graphs. Here we define a *complete bipartite graph K*_*m*,*n*_ as a bipartite graph where |*V*_1_| = *m*, |*V*_2_| = *n* and each vertex in *V*_1_ is connected with each vertex in *V*_2_. A graph *H*(*W*, *F*) is a *subgraph* of *G*(*V*, *E*) if 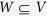 and 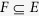. It is possible that the interaction graph of the desired Ising Hamiltonian is a subgraph of the hardware connectivity graph. In this case the embedding problem can be solved by *subgraph embedding*, which we define as the following.

**Definition 3.** *A* subgraph embedding *of G*(*V*, *E*) *into G*′(*V*′, *E*′) *is a mapping* 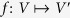 *such that each vertex in V is mapped to a unique vertex in V*′ *and if* 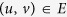 *then* 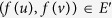.

In more general cases, for an arbitrary Ising Hamiltonian, a subgraph embedding may not be obtainable and we will need to embed the interaction graph into the hardware as a graph *minor*. Before we define minor embedding rigorously, recall that a graph is *connected* if for any pair of vertices *u* and *v* there is a path from *u* to *v*. A *tree* is a connected graph which does not contain any simple cycles as subgraphs. *T* is a *subtree* of *G* if *T* is a subgraph of *G* and *T* is a tree. We then define minor embedding as the following.

**Definition 4.** *A* minor embedding *of G*(*V*, *E*) *in G*′(*V*′, *E*′) *is defined by a mapping* 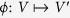 *such that each vertex* 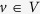 *is mapped to a connected subtree T*_*v*_ *of G*′ *and if* 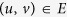 *then there exist i*_*u*_, 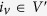 *such that* 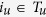, 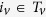 *and* 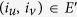.

If such a mapping *ϕ* exists between *G* and *G*′, we say *G* is a *minor* of *G*′ and we use *G* ≤ _*m*_*G*′ to denote such relationship. Our goal is to take the interaction graph *G*_*H*_ of our Ising Hamiltonian construction and construct the mapping *ϕ* that embeds *G*_*H*_ into the hardware graph as a minor.

### Chimera graphs

**Figure 1:**
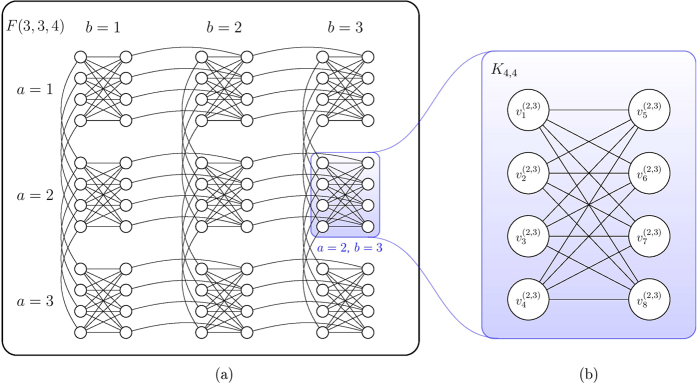
The Chimera graph that represents the qubit connectivity of D-Wave hardware. (**a**) Example of a 3 × 3 grid of *K*_4,4_ cells, denoted as *F*(3, 3, 4). (**b**) Labelling of nodes within a particular cell on the *a*-th row and *b*-th column. Here we use the cell on the 2nd row and 3rd column as an example.

Here we specifically consider the embedding our construction into a particular type of hardware graphs used by D-Wave devices^44^,^65^ called the *Chimera graphs*. The basic components of this graph are 8-spin unit cells^6^ whose interactions form a *K*_4,4_. The *K*_4,4_ unit cells are tiled together and the 4 nodes on the left half of *K*_4,4_ are connected to their counterparts in the cells above and below. The 4 nodes on the right half of *K*_4,4_ are connected to their counterparts in the cells left and right. Furthermore, we define *F*(*p*, *q*, *c*) as a Chimera graph formed by an *p* × *q* grid of *K*_*c*,*c*_ cells. Figure 1a shows *F*(3, 4) as an example. Note that any *K*_*m*,*n*_ with *m*, *n* ≤ *c* can be trivially embedded in *F*(*p*, *q*, *c*) with any *p*, *q* ≥ 1 via subgraph embedding. However, it is not clear *a priori* how to embed *K*_*m*,*n*_ with *m* > *c* or *n* > *c* onto a Chimera graph, other than using the general embedding of an (*m* + *n*)-node complete graph and consider *K*_*m*,*n*_ as a subgraph. This costs *O*((*m* + *n*)^2^) qubits in general and one may lose the intuitive structure of a bipartite graph in the embedding. One of the building blocks of our embedding for our Ising Hamiltonian construction (Section Embedding on quantum hardware) is an alternative embedding strategy for mapping any *K*_*m*,*n*_ onto 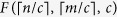 as a graph minor. Our embedding costs *O*(*mn*) qubits and preserves the structure of the bipartite graph.

## Quantum annealing for solving SCP

### From an arbitrary SCP instance to an Ising Hamiltonian construction

SCP is NP-complete most simply because Set Cover (SC) is a special case of SCP^56^ and a solution to SCP is clearly efficiently verifiable. Since SC is NP-complete itself, any SCP instance can be rewritten as an instance of SC with polynomial overhead. The Ising Hamiltonian construction for Set Cover is explicitly known^39^,^50^. Hence it is natural to consider using the chain of reductions from SCP to SC and then from SC to ISING (Definition 2). If we recast each SCP(*G*, *U*, *S*) with |*S*| = *m* into an SC instance with a cover set of size *O*(*m*^2^). Using the construction by Lucas^50^ we have an Ising Hamiltonian


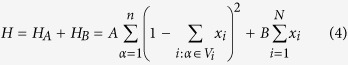


where *V*_*i*_ is the *i*-th cover set in the SC instance. Since the cover set {*V*_*i*_} is possibly of size up to *O*(*m*^2^), this leads to the Ising Hamiltonian in equation (4) costing *O*(*nm*^2^) qubits.

Here we present an alternative Ising Hamiltonian construction for encoding the solution to any SCP instance. We state the result precisely as Theorem 1 below. The qubit cost of our construction is comparable to that of Lucas. However, in Section Embedding on quantum hardware we argue that our construction affords more advantages in terms of embedding.

**Theorem 1.** *Given an instance of the Set Cover with Pairs Problem* SCP(*G*, *U*, *S*) *as in Definition* 1, *there exists an efficient* (*classical*) *algorithm that computes an instance of the Ising Hamiltonian ground state problem* ISING(**h**, **J**) *with* 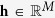 *and* 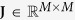 *where the number of qubits involved in the Hamiltonian is M* = *O*(*nm*^2^) *with n* = |*U*| *and m* = |*S*|.

*Proof*. First, we recast an SCP instance to an instance of integer programming, which is NP-hard in the worst case. Then, we convert the integer programming problem to an instance of the ISING problem. Recall Definition 1 of an SCP(*G*, *U*, *S*) instance, where *G*(*V*, *E*) is a graph on the vertices *V* = *U* ∪ *S*. For each pair 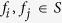 define a set 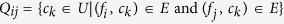. The problem can be recast as an integer program by


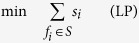



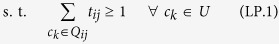



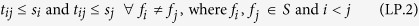



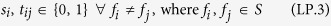


We have introduced the variable *s*_*i*_ to indicate whether *f*_*i*_ is chosen for the cover *A* ⊆ *S* (*s*_*i*_ = 1 means that *f*_*i*_ is chosen, otherwise *s*_*i*_ = 0). We have also introduced the auxiliary variable *t*_*ij*_ to indicate whether *f*_*i*_ and *f*_*j*_ are *both* chosen. Hence, constraint LP.1 ensures that each element *c*_*k*_ ∈ *U* is covered by at least one pair in *S*. LP.2 ensures that a pair of elements in *S* cannot cover any *c*_*k*_ ∈ *U* unless both elements are chosen.

To convert the integer program to an ISING instance, we first convert the constraints into expressions of logical operations. LP.1 can be rewritten as


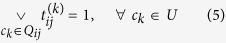


LP.2 can be translated to a truth table for the binary operation involving *t*_*ij*_ and *s*_*i*_(*s*_*j*_) where only the entry 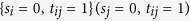 evaluates to 0 and the other three entries evaluate to 1. Using the following Hamiltonians we could translate the logic operations ∨, ∧ and ≤ into the ground states of Ising model, see ref. 66 for more details.


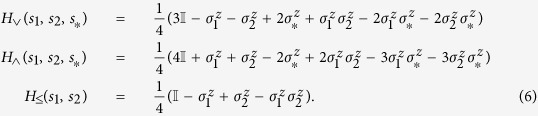


Note that *H*_≤_(*s*_1_, *s*_2_) is essentially 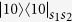. In other words we are penalizing the only 2-bit string *s*_1_*s*_2_ that violates the constraint *s*_1_ ≤ *s*_2_. The ground state subspace of *H*_∨_ is spanned by 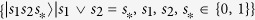. Similarly, the ground state subspace of *H*_∧_ is spanned by 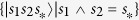 and that of *H*_≤_ spanned by 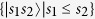.

By linearly combining the above constraint Hamiltonians, we can enforce multiple constraints to hold at the same time. For example, the statement 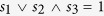 can be decomposed as simultaneously ensuring 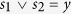, 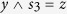, and *z* = 1. In other words we have used auxiliary variables *y* and *z* to transform the constraint 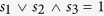, which involves a clause 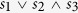 of three variables, to a set of constraints involving only clauses of two variables. Then, the Ising Hamiltonian 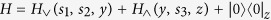 has its ground state spanned by states 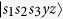 with *s*_1_, *s*_2_, and *s*_3_ satisfying 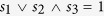. The third term in *H* ensures that *z* = 1 by penalizing states with 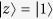.

Therefore, we can translate (5) to an Ising Hamiltonian. For a fixed *k*, the constraint (5) takes the form of 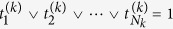 where each 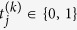 and 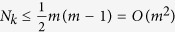. Similarly to the example above, we introduce *N*_*k*_ − 1 auxiliary variables 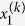, 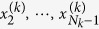 such that


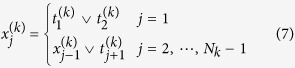


Thus, 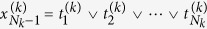. In order to ensure the first constrain holds, it is needed to ensure that 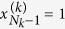. Then we could write down the corresponding Ising Hamiltonian for the constraint as


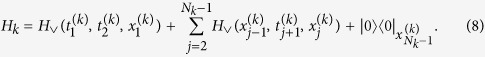


The last term is meant to make sure that 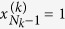 in the ground state of *H*_*k*_. Therefore the Hamiltonian whose ground state subspace is spanned by all states that obey both of the constraints in the integer program (5) can be written as


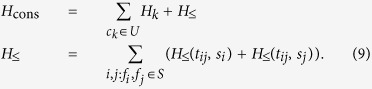


The target function 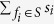 which we seek to minimize can be directly mapped to an Ising Hamiltonian 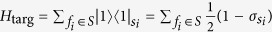. This is because we would like to essentially minimize the number of 1’s in the set of *s*_*i*_ values and penalize choices with more 1’s. Therefore the final Hamiltonian whose ground state contains the solution to the original SCP instance becomes


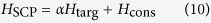


for some weight factor *α*.

We now estimate the overhead for the mapping. *H*_targ_ acts on |*S*| = *m* qubits. In *H*_cons_, *H*_≤_ acts on *O*(*m*^2^) qubits, since there are *O*(*m*^2^) variables *t*_*ij*_. Each *H*_*k*_ in *H*_cons_ requires *N*_*k*_ = *O*(*m*^2^) qubits. There are in total |*U*| = *n* of the *H*_*k*_ terms, which gives *O*(*nm*^2^) qubits in total. □

#### Example

**Figure 2:**
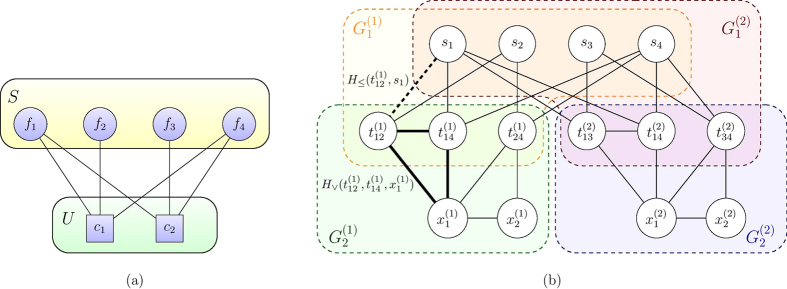
Example of converting an SCP instance to Ising Hamiltonian. (**a**) The SCP instance. Here 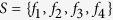 and 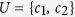. The solution is the set 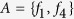. The circles represent the covering set elements *S* and the squares are the ground elements *U*. (**b**) The interaction of Ising instance *H*_SCP_ converted from the SCP instance in (**a**). Every node corresponds to a qubit. The *s*_*i*_’s are the output bits that correspond to the covering set elements *S*. The others are auxiliary variables. Every edge represents an interaction term between the corresponding spins. Here we do not show the 1-local terms in our construction of *H*_SCP_ (for example the terms in *H*_targ_ for enforcing the minimization of the target function). The bold dashed black line exemplifies the edges between the 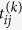 nodes and the *s*_*i*_ nodes, which come from the constraints 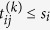 and 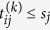 for each pair 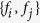 that covers *c*_*k*_. Each of the inequality constraints is enforced by a *H*_≤_ term in (6). The bold triangle exemplifies the *H*_∨_ constraints in (6) that are used to enforce the logical relationship between the 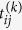 variables and the auxiliary variables as shown in (7). The areas marked by 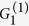, 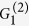 etc outline the structure of the Ising Hamiltonian that is relevant in the discussion of hardware embedding.

Consider the SCP instance shown in Fig. 2a. With the mapping presented in Theorem 1, we arrive at an Ising instance ISING (**h**, **J**) where *α* = 1/4 in (10) and **h**, **J** are presented in Supplementary Material Details of the example SCP instance. The ground state subspace of the Hamiltonian in (2) with *h*_*i*_ and *J*_*ij*_ coefficients defined above, restricted to the *s*_*i*_ elements is spanned by 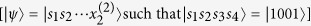. This corresponds to *A* = {*f*_1_, *f*_4_}, the solution to the SCP instance. Figure 2b illustrates the interaction graph of the spins in the Ising Hamiltonian that corresponds to the SCP instance.

### Numerical simulation of quantum annealing

In order to test the time complexity of using quantum annealing to solve SCP instances via the construction in Theorem 1, we generate random instances of SCP that lead to Ising Hamiltonian *H*_*SCP*_ of 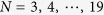 spins. In Definition 1 we use a bipartite graph between the ground state *U* of size *n* and the cover set *S* of size *m* to describe an SCP instance. For fixed *n* and *m*, there are in total 2^*mn*^ such possible bipartite graphs (if we consider each bipartite graph as a subgraph of *K*_*m*,*n*_ and count the cardinality of the power set of the edges of *K*_*m*,*n*_). Therefore to generate random bipartite graphs we only need to flip *mn* fair coins to uniformly choose from all possibile bipartite graphs between *U* and *S*. However, we would like to exclude the bipartite graphs where some element of *S* is not connected to any element in *U*. These “dummy nodes” are not pertinent to the computational problem at hand and should be removed from consideration before converting the SCP instance to an Ising Hamiltonian *H*_SCP_. We thus use a scheme for generating random instances of SCP *without* dummy nodes as described in Algorithm 1. Under the constraint that no dummy element in *S* is allowed, there are in total (2^*n*^ − 1)^*m*^ possible bipartite graphs. In Supplementary Material Proof of correctness for Algorithm 1 we rigorously show that Algorithm 1 indeed samples uniformly among the (2^*n*^ − 1)^*m*^ possible “dummy-free” bipartite graphs.


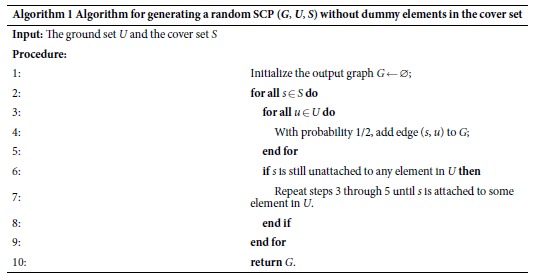


For each randomly generated instance from Algorithm 1 we construct an Ising Hamiltonian *H*_*SCP*_ according to Theorem 1. We then perform a numerical simulation of the time dependent Schrödinger equation (1) from time *t* = 0 to *t* = *T* with time step Δ*t* = 1 and the time dependent Hamiltonian defined as


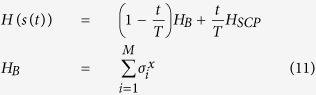


where *H*_SCP_ is defined in equation (10). Here because of the construction of *H*_SCP_, our total Hamiltonian *H*(*s*(*t*)) acts not only on the spins 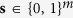 indicating our choice of elements in the cover set *S*, but also auxiliary variables 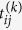 and 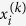, for which we use **t** and **x** to denote their respective collections. Our initial state is the ground state of *H*_*B*_, namely


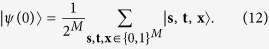


**Figure 3:**
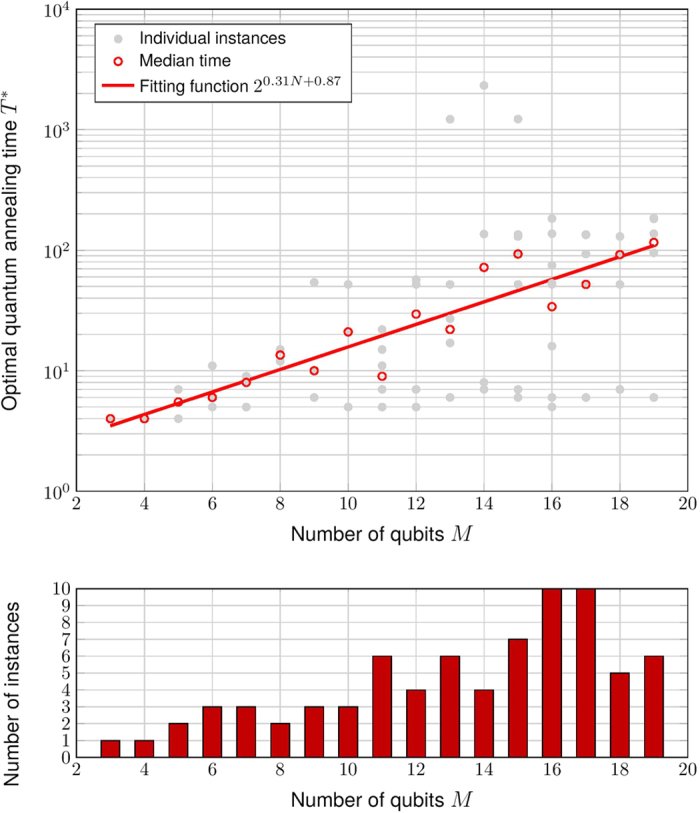
Plot of the optimal quantum annealing time T* versus the number of spins involved in the construction of *H*_SCP_. Here we fit the logarithm of median T* with a straight line. The size *M* of our Ising systems ranges from 3 to 19. From the fitting function we observe that the annealing time scales as roughly *O*(2^0.31*M*^). We also provide on the bottom plot the number of instances for each *M*.

To obtain the final state 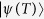 where *T* is some positive integer, we use the ode45 subroutine of MATLAB under default settings to numerically integrate Schrödinger equation to obtain 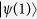 from 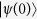, and then use 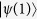 as an initial state to obtain 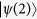 in the same fashion, and so on. We define the success probability *p* as a function of the total annealing time *T* as 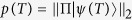 where Π is a projector onto the subspace spanned by states with *s* being a solution of the original SCP instance. Using binary search we determine the minimum time T* to achieve 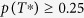 for each instance of SCP. Figure 3 shows the distribution of T* for SCP instances that lead to Ising Hamiltonians *H*_SCP_ of the same sizes, as well as how the median annealing time scales as a function of number of spins *M*. Results show that for instances with *M* up to 19, the median annealing time scales roughly as *O*(2^0.31*M*^).

### Numerical experiment with Simulated Annealing

Simulated annealing, first introduced three decades ago^67^, has been widely used as a heuristic for handling hard combinatorial optimization problems. It is especially of interest as a benchmark for quantum annealing^34^,^35^,^36^ because of similarities between the two algorithms. While quantum annealing employs quantum tunneling to escape from local minima, simulated annealing relies on thermal excitation to avoid being trapped in local minima. The general procedure we adopt for simulated annealing to approach the ground state of an Ising spin glass can be summarized as the following^68^:

1. Repeat *R* times the following:

- Initialize **s** ← *s*_0_ randomly;
- Perform *S* times the following: (let 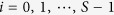 index the steps)

- Set the temperature 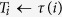;
-  Perform a *sweep* on **s**_*i*_ to obtain **s**′; (a sweep is a sequence of steps each of which randomly selects a spin and flips its state, so that on average each spin is flipped once during a sweep)
-  With probability 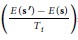, let 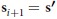. Otherwise let 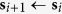.

1. Return **s**_*S*_ as the answer.

For the purpose of comparison we also used simulated annealing to solve the same set of instances generated by Algorithm 1 for testing quantum annealing. The program implementation that we use is built by Isakov *et al*.^68^, which is a highly optimized implementation of simulated annealing with care taken to exploit the structures of the interaction graph, such as being bipartite and of bounded degree. Here we use the program’s most basic realization of single-spin code for general interactions with magnetic field on an interaction graph of any degree.

As mentioned by Isakov *et al*., to improve the solution returned by simulated annealing, one could increase either the number of sweeps *S* or number of repetitions *R* in the implementation, or both of them. However, note that the total annealing time is proportional to the product 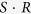 and there is a trade-off between *S* and *R*. For a fixed number of sweeps *S* let the success probability (*i*.*e*. the fraction of **s**_*i*_ that is satisfactory) be *w*(*S*). In order to achieve a constant success probability *p* (say 25%, which is what we use here), we need at least 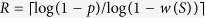 repetitions. Hence the total time of simulated annealing can be written as


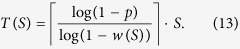


**Figure 4:**
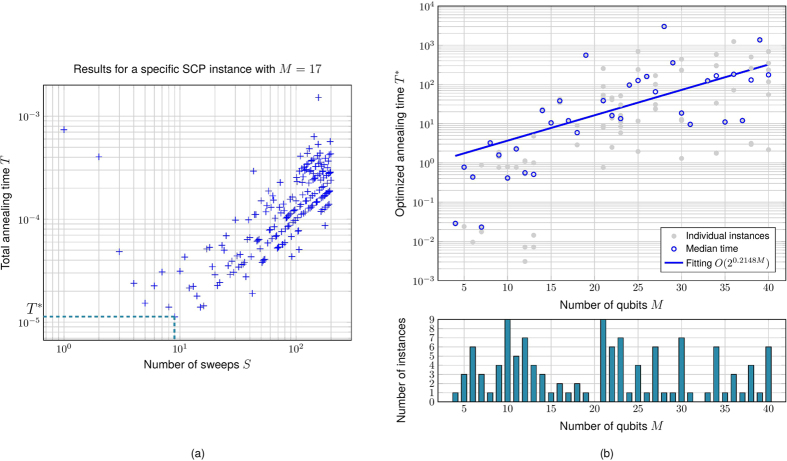
(**a**) Plot of annealing time *T* versus number of sweeps *S* using the simulated annealing implementation^68^ on an Ising Hamiltonians of 17 spins constructed from an SCP instance. We use the default settings for all parameters other than *S* and *R*. Also we mark the optimal runtime T_*_. (**b**) Plot of optimized annealing time T* versus the number of spins involved in the Ising Hamiltonian HSCP corresponding to randomly generated SCP instances according to Algorithm 1. We also provide on the bottom plot the number of instances for each *M*.

In general *w*(*S*) increases as *S* increases, leading to a decrease in *R*. We numerically investigate this with an Ising system of *N* = 17 spins generated from an SCP instance via the construction in Theorem 1. We plot the annealing time *T* versus *S* in Fig. 4a. For each SCP instance with the number of spin *M* we compute the optimal S* such that 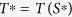 is the optimized runtime (Fig. 4a). We further explore how the optimal runtime T* scales as a function of the number of spins *M*. As shown in Fig. 4b, a linear fit on a semilog plot shows that roughly 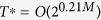.

The units of time used for both Fig. 4a,b are arbitrary and thus do not support a point-to-point comparison. But the scaling difference seems apparent. For quantum annealing we restrict to systems of at most 19 spins due to computational limitations faced in representing the full Ising Hamiltonian when numerically integrating the time-dependent Schrödinger equation (1).

Although there is no quantum speedup observed in terms of median runtime over all randomly generated instances of the same size, we notice that for a fixed number of spins *M* the performances of both quantum annealing and simulated annealing are sensitive to the specific instance of Ising Hamiltonian *H*_SCP_ than simulated annealing. This can be seen by considering at the same time the quantum annealing results in Fig. 3 and the test results for simulated annealing shown in Fig. 4b. One could then speculate that perhaps by focusing on a specific subset of SCP instances could yield a quantum advantage.

## Embedding on quantum hardware

In this section we deal with the physical realization of quantum annealing for solving SCP instances using D-Wave type hardwares. There are mainly two aspects^62^,^69^ of this effort: 1) The *embedding problem*^62^, namely embedding the interaction graph of the Ising Hamiltonian construction *H*_SCP_ as a graph minor of a Chimera graph (refer to Section Graph minor embedding for definitions of the graph terminologies). 2) The *parameter setting problem*^69^, namely assigning the strengths of the couplings and local magnetic fields for embedded graph on the hardware, in a way that minimizes the energy scaling (or control precision) required for implementing the embedding. Here we focus on the former issue.

We start with an observation on the structures of *H*_SCP_. For any instance SCP(*G*, *U*, *S*) according to Definition 1, the interaction graph *I*_SCP(*G,U,S*)_ of the corresponding Ising Hamiltonian *H*_SCP_ can be regarded as a union of *n* subgraphs, namely 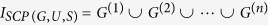. Each subgraph *G*^(*i*)^ is associated with an element of the ground set 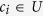 as in Fig. 2a. Each *G*^(*i*)^ could be further partitioned into two parts, 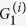 and 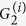. For any *k*, 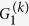 is a bipartite graph between 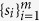 and 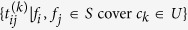. 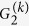 essentially describes the interaction between the auxiliary variables 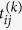 and 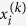 as described in equation (7). In Fig. 2b we illustrate such partition using the example from Fig. 2b. Our goal is then to show constructively that *I*_SCP_(G,U,S) ≤ _*m*_*F*(*f*_1_, *f*_2_, *c*) for some *f*_1_, *f*_2_ that depend on *m*, *n* and *c* = 4, which describes the Chimera graph realized by D-Wave hardware (Fig. 1a).

It is known^61^ that one could embed a complete graph on *cm* + 1 nodes onto Chimera graph 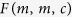. Since any *n*-node graph is a subgraph of the *n*-node complete graph, in principle any *n*-node graph can be embedded onto Chimera graphs of size *O*(*n*^2^) using the complete graph embedding. A downside of this approach is that it may fail to embed many graphs that are in fact embeddable^61^. Also, using embeddings based on complete graph embeddings will likely lose the intuition on the structure of the original graph. For graphs with specific structures, such as bipartite graphs one may be able to find an embedding that is also in some sense structured. We show in the following Lemma an embedding for any complete bipartite graph *K*_*p*,*q*_ onto a Chimera graph. The ability to do so enables us to embed any bipartite graph onto a Chimera graph.

**Lemma 1.** *For any positive integers p*, *q and c*, 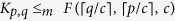.

*Proof*. By the definition of graph minor embedding in Section Graph minor embedding, it suffices to construct a mapping 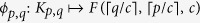 where each *v* in *F*_*p*,*q*_ is mapped to a tree *T*_*v*_ in 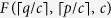 and each edge *e* = (*u*, *v*) in *K*_*p*,*q*_ is mapped to an edge 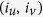 with 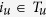 and 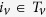.

Let 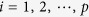 label the nodes on one side of *K*_*p*,*q*_ and 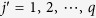 label the nodes in the other. Using the labelling scheme on the nodes of Chimera graphs introduced in Section Chimera graphs and Fig. 1b, we define our mapping 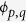 as


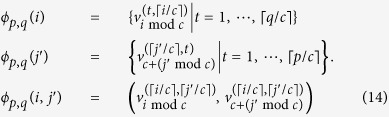


where 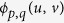 maps an edge (*u*, *v*) in *K*_*p*_,_*q*_ to the Chimera graph. If we choose the edges in the Chimera graph properly, it could be checked that 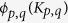 is a subgraph of 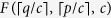.□

**Figure 5:**
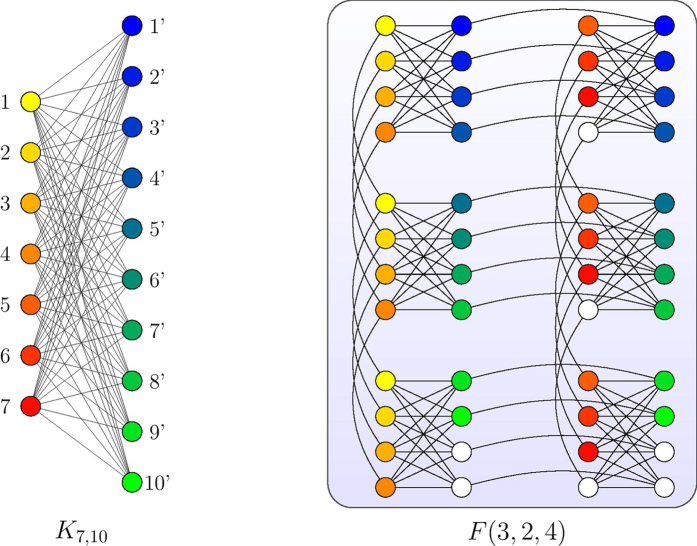
An example showing the embedding scheme outlined in Lemma 1. The nodes and the trees mapped from the nodes are marked with the same colors.

In Fig. 5 we show an example of embedding *K*_7,10_ into *F*(3, 2, 4). A natural corollary of Lemma 1 is that any bipartite graph between *p* and *q* nodes can be minor embedded in 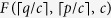. We are then prepared to handle embedding the 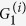 parts of the interaction graphs of *H*_SCP_, which are but bipartite graphs (see Fig. 2b for example).

We then proceed to treat the 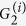 parts of the interaction graph. The connectivity of 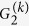 is completely specified by (7). To describe such connectivity we define a family of graph 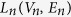 as 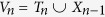 where 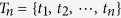 and 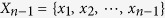 are two disjoint sets of nodes, the former representing the intermediate variables 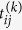 and the latter representing the *x*_*k*_ variables in equation (7). The set of edges takes the form


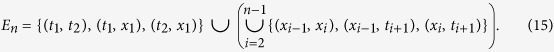


**Figure 6:**
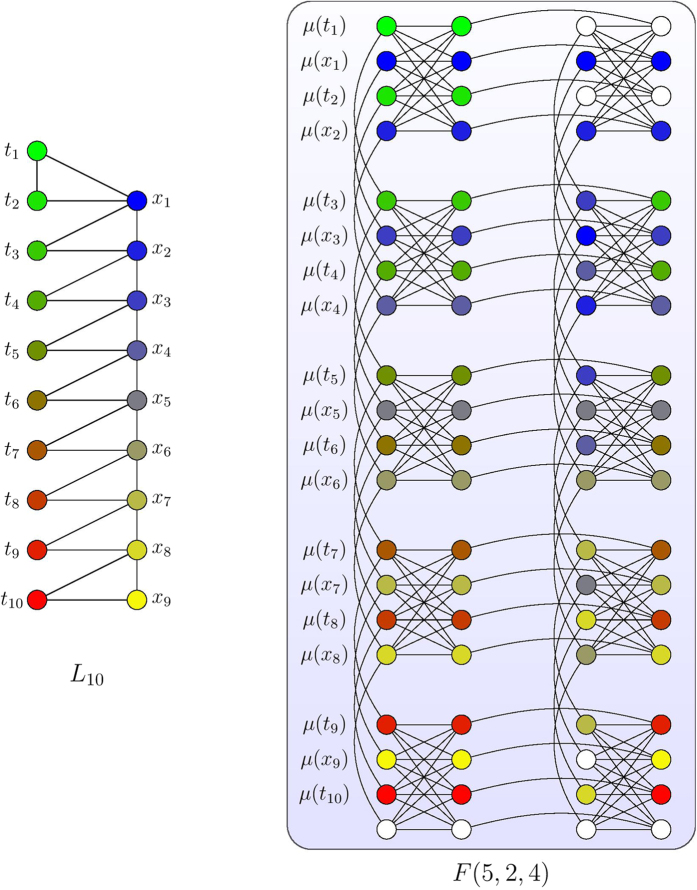
An example of embedding *L*_10_ onto *F*(5, 2, 4). Each color in the left diagram represents a node *u* in *L*_10_ and the nodes of the same color in the right diagram shows *μ*_10_(*u*).

In Fig. 6 we show an example of *L*_10_. For any 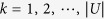, let *r*_*k*_ be the number of pairs 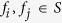 that cover *k*. Then 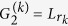. Hence in order to show that we could embed any 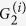 onto a Chimera graph, it suffices to show that we can embed any *L*_*n*_ onto a Chimera graph. We show this in the following Lemma for *c* = 4.

**Lemma 2.** *For any positive integer n*, 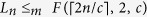 *where we restrict to c* = 4.

*Proof*. Similar to Lemma 1, we construct a mapping 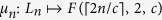 where we fix *c* = 4. Following the notation for nodes in *L*_*n*_ in Fig. 6 and the notation for nodes in 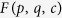 in Fig. 1b, we construct *μ* as


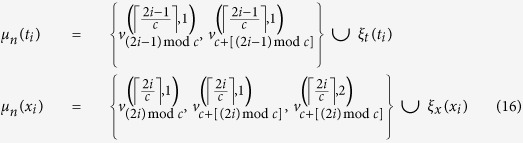


where 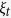 and 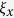 are defined as


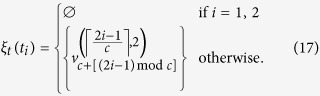



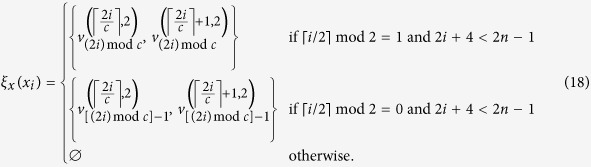


With the vertex mapping *μ*_*n*_, a mapping of edges in *L*_*n*_ onto the Chimera graph 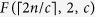 is easy to find.

In Fig. 6 we show an example of embedding *L*_10_ onto *F*(5, 2, 4). We could then proceed to embed the interaction graph *I*_SCP(*G*,*U*,*S*)_, such as the one shown in Fig. 2b, in a Chimera graph. Specifically, we state the following theorem.

**Theorem 2.** *For any instance* 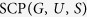 *with* 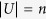 *and* 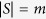, 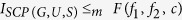 *where* 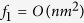, 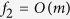 *and c* = 4 *is a constant*.

**Figure 7:**
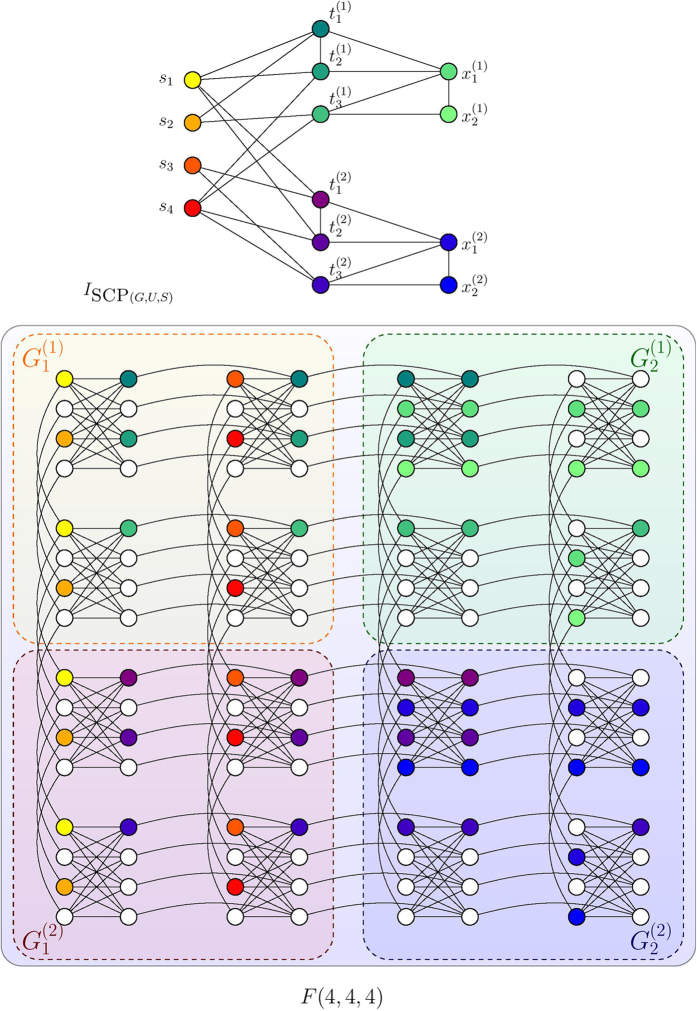
Embedding the interaction graph of the example physical system in Fig. 2b onto *F*(4, 4, 4). Note that the structure of Fig. 2 is preserved on the Chimera graph.

*Proof*. Our embedding combines ideas from Lemma 1 and 2. We modify the mapping *ϕ*_*p*_,_*q*_ constructed in Lemma 1 to produce a new mapping *θ*_*p*,*q*_ that produces more spacing between the embedded nodes (see for example 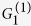 and 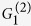 in Fig. 7):


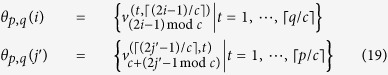


Let 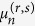 denote a mapping *μ* described in Lemma 2 that maps the upper left node (Fig. 6) *t*_1_ to 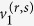 instead of 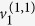. The rest of the mapping then proceeds from 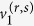. In other words, 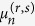 is the mapping *μ* that is shifted by *p* − 1 cells to the right and *q* − 1 cells below. Trivially 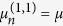. Similarly we define 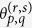 as the shifted embedding under *θ*_*p*,*q*_ where 
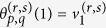
. Recall that for any ground set element 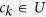, *r*_*k*_ is the number of pairs in *S* that covers *c*_*k*_. We could then specify the embedding from 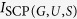 onto 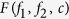 as


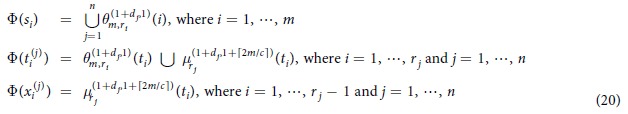


where 
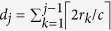
 is the total number of rows of cells occupied by the embedded graphs for handling the ground elements *c*_1_ through *c*_*j*−1_. In total Φ(*I*_SCP(*G*,*U*,*S*)_) will occupy 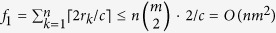 rows and 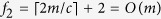 columns.□

In Fig. 7 we show an embedding 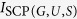 of the example instance in Fig. 2 onto *F*(4, 4, 4). Note that our embedding preserves the original structure of the interaction graph as shown in Fig. 2b. Furthermore, note that the interaction graph *I*_SCP(*G*,*U*,*S*)_ has 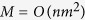 nodes. Using the complete graph embedding requires 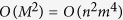 qubits. For the same reason, the construction of Ising Hamiltonian described in equation (4) is likely going to cost *O*(*nm*^4^) in the worst case of embeding in a Chimera graph since the interaction graph of the Hamiltonian could involve complete graphs of size *O*(*m*^2^) due to the square term *H*_*A*_. By comparison our embedding costs 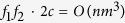 qubits and preserves the structure of the original instance, which affords slightly more advantage for scalable physical implementations.

## Discussion

Our interest in SCP is largely motivated by its important applications in various areas^57^,^58^,^59^,^60^. We have shown a complete pipeline of reductions that converts an arbitrary SCP instance to an interaction graph on a D-Wave type hardware based on Chimera graphs, in a way that preserves the structure of the instance throughout (Figs 2b and 7) and is more qubit efficient than the usual approach by complete graph embedding. Although no quantum speedup is observed at this stage based on comparison of median annealing times, the large variance of runtimes observed in Fig. 3a from instance to instance might suggest that specific subsets of instances could provide quantum speedup. Of course, a clearer understanding of the performance of quantum annealing on solving SCP could only be brought forth by both scaling up the numerical simulation of the quantum annealing process to include instances with larger number of spins and actual experimental implementation of the quantum annealing process. Both of them are of interest to us in our future work.

## Additional Information

**How to cite this article**: Cao, Y. *et al*. Solving Set Cover with Pairs Problem using Quantum Annealing. *Sci. Rep*. **6**, 33957; doi: 10.1038/srep33957 (2016).

## Supplementary Material

Supplementary Information
