# Supplementary material for: Solving Set Cover with Pairs Problem using Quantum Annealing
Source: Sci Rep. 2016 Sep 27;6:33957. doi: 10.1038/srep33957 (PMC5037405; doi:10.1038/srep33957)
Supplement: Supplementary Information [file srep33957-s1.pdf]

Supplementary online material for “Solving Set Cover with Pairs Problem using quantum annealing”

Yudong Cao*[1], Shuxian Jiang[1], Debbie Perouli[2], and Sabre Kais[3,4]

[1]Department of Computer Science, Purdue University, West Lafayette, IN 47906, USA
[2]Department of Mathematics, Statistics and Computer Science, Marquette University, Milwaukee, WI 53233, USA
[3]Department of Chemistry, Physics and Birck Nanotechnology Center, Purdue University, West Lafayette, IN 47906, USA
[4]Qatar Energy and Environment Research Institute (QEERI), HBKU, Doha, Qatar

Details of the example SCP instance

In the paper we consider an example SCP instance for illustrating our mappings from SCP to Ising and eventually to a Chimera graph. Specifically, the $\text{ISING}(\mathbf{h},\mathbf{J})$ described in Figure 2b has

$$\mathbf{h}^T = \frac{1}{8}\cdot \begin{pmatrix} \overset{s_1}{7} & \overset{s_2}{3} & \overset{s_3}{3} & \overset{s_4}{7} & \overset{t_{12}^{(1)}}{-6} & \overset{t_{14}^{(1)}}{-6} & \overset{t_{24}^{(1)}}{-6} & \overset{t_{13}^{(2)}}{-6} & \overset{t_{14}^{(2)}}{-6} & \overset{t_{34}^{(2)}}{-6} & \overset{x_1^{(1)}}{2} & \overset{x_2^{(1)}}{4} & \overset{x_1^{(2)}}{2} & \overset{x_2^{(2)}}{4} \end{pmatrix}.$$

Here the labels above each element of $\mathbf{h}$ indicates which spin the coefficient is associated to. The matrix of interaction coefficients $\mathbf{J}$ is shown in Figure S1.

Proof of correctness for Algorithm 1

Here we show that Algorithm 1 indeed samples uniformly from all $(2^n-1)^m$ possible “dummy-free” bipartite graphs for a fixed setting of the ground set U of size n and cover set S of size m. Formally we say a bipartite graph $G(U\cup S,E)$ between two sets U and S is *dummy-free* if for any $s\in S$ there exists at least one $u\in U$ such that $(s,u)\in E$. Then we state the following claim.

Claim. *Given any set U of n elements and S of m elements, for any dummy-free bipartite graph $G(V,E)$ between U and S, Algorithm 1 generates G with probability $(2^n-1)^{-m}$.*

Proof. Let $\Pr(G)$ be the probability that Algorithm 1 generates G. Recall that if at a particular $s\in S$ during the looping on line 2, when Algorithm 1 scanned through all $u\in U$ but did not end up selecting any element in U, the algorithm enters line 7 to repeat the process from scratch for s. Then depending on how many times the algorithm entered line 7 during the process of generating G, we could express $\Pr(G)$ as

$$\Pr(G)=\sum_{k=0}^{\infty}\Pr(G|\text{Algorithm 1 entered line 7 in total } k \text{ times}) \tag{S2}$$

If the algorithm never entered line 7 and generated G, then the probability of generating G is essentially the probability of mn coin flips, namely 2^{-mn}. If the algorithm entered line 7 once, then the probability $\Pr(G)=2^{-mn}\cdot m2^{-n}$, where the extra factor $m2^{-n}$ is essentially the probability of one hit and $m-1$ misses during m

*cao23@purdue.edu

$$
\mathbf{J} = \frac{1}{8} \cdot \left(\begin{array}{c|cccccccccccccc}
 & s_1 & s_2 & s_3 & s_4 & t_{12}^{(1)} & t_{14}^{(1)} & t_{24}^{(1)} & t_{13}^{(2)} & t_{14}^{(2)} & t_{34}^{(2)} & x_1^{(1)} & x_2^{(1)} & x_1^{(2)} & x_2^{(2)} \\
\hline
s_1 & & & & & -1 & -1 & & -1 & -1 & & & & & \\
s_2 & & & & & -1 & & -1 & & & & & & & \\
s_3 & & & & & & & & -1 & & -1 & & & & \\
s_4 & & & & & & -1 & -1 & & -1 & -1 & & & & \\
t_{12}^{(1)} & -1 & -1 & & & & 1 & & & & & -2 & & & \\
t_{14}^{(1)} & -1 & & & -1 & 1 & & & & & & -2 & & & \\
t_{24}^{(1)} & & -1 & & -1 & & & & & & & 1 & -2 & & \\
t_{13}^{(2)} & -1 & & -1 & & & & & & 1 & & & & -2 & \\
t_{14}^{(2)} & -1 & & & -1 & & & & 1 & & & & & -2 & \\
t_{34}^{(2)} & & & -1 & -1 & & & & & & & & & 1 & -2 \\
x_1^{(1)} & & & & & -2 & -2 & 1 & & & & & -2 & & \\
x_2^{(1)} & & & & & & & -2 & & & & -2 & & & \\
x_1^{(2)} & & & & & & & & -2 & -2 & 1 & & & & -2 \\
x_2^{(2)} & & & & & & & & & & -2 & & & -2 &
\end{array}\right) \tag{S1}
$$

Figure S1: The matrix of coupling coefficients in the Ising Hamiltonian instance constructed for the example SCP instance shown in Figure 2. The interpretation of the matrix elements of $\mathbf{J}$ follows Definition 2.

independent Bernoulli trial with the hit probability 2^{-n} (if we regard the event of entering line 7 as a hit). Carrying this argument to the general case if the algorithm enters line 7 k times, then we need to consider all possible ways of distributing the k hits onto the m iterations on line 2. This gives

$$
\Pr(G|\text{Algorithm 1 entered line 7 in total } k \text{ times}) = \sum_{(k_1,\cdots,k_m)} 2^{-mn} \cdot \binom{k}{k_1, k_2, \cdots, k_m} \cdot (2^{-n})^{k_1+k_2+\cdots+k_m} \tag{S3}
$$

where the summation is over the set of non-negative integers k_1 through k_m that sums up to k. Then Equation S2 leads to

$$
\begin{aligned}
\Pr(G) &= 2^{-mn} \cdot \sum_{k=0}^{\infty} \sum_{(k_1,\cdots,k_m)} 2^{-kn} \binom{k}{k_1, \cdots, k_m} \\
&= 2^{-mn}(1 + 2^{-n} + 2^{-2n} + \cdots)^m \\
&= 2^{-mn} \left(\frac{1}{1 - 2^{-n}} \right)^m \\
&= (2^n - 1)^{-m}.
\end{aligned} \tag{S4}
$$

□
